# Exploring the influence of environmental indicators and forecasting influenza incidence using ARIMAX models

**DOI:** 10.3389/fpubh.2024.1441240

**Published:** 2024-09-23

**Authors:** Xiaoyan Zheng, Qingquan Chen, Mengcai Sun, Quan Zhou, Huanhuan Shi, Xiaoyang Zhang, Youqiong Xu

**Affiliations:** ^1^The Affiliated Fuzhou Center for Disease Control and Prevention of Fujian Medical University, Fuzhou, China; ^2^The School of Public Health, Fujian Medical University, Fuzhou, China

**Keywords:** influenza incidence, air pollution, meteorological factor, time series, ARIMAX model, prediction influenza incidence, prediction

## Abstract

**Background:**

Influenza is a respiratory infection that poses a significant health burden worldwide. Environmental indicators, such as air pollutants and meteorological factors, play a role in the onset and propagation of influenza. Accurate predictions of influenza incidence and understanding the factors influencing it are crucial for public health interventions. Our study aims to investigate the impact of various environmental indicators on influenza incidence and apply the ARIMAX model to integrate these exogenous variables to enhance the accuracy of influenza incidence predictions.

**Method:**

Descriptive statistics and time series analysis were employed to illustrate changes in influenza incidence, air pollutants, and meteorological indicators. Cross correlation function (CCF) was used to evaluate the correlation between environmental indicators and the influenza incidence. We used ARIMA and ARIMAX models to perform predictive analysis of influenza incidence.

**Results:**

From January 2014 to September 2023, a total of 21,573 cases of influenza were reported in Fuzhou, with a noticeable year-by-year increase in incidence. The peak of influenza typically occurred around January each year. The results of CCF analysis showed that all 10 environmental indicators had a significant impact on the incidence of influenza. The ARIMAX(0, 0, 1) (1, 0, 0)_12_ with PM_10_(lag5) model exhibited the best prediction performance, as indicated by the lowest AIC, AICc, and BIC values, which were 529.740, 530.360, and 542.910, respectively. The model achieved a fitting RMSE of 2.999 and a predicting RMSE of 12.033.

**Conclusion:**

This study provides insights into the impact of environmental indicators on influenza incidence in Fuzhou. The ARIMAX(0, 0, 1) (1, 0, 0)_12_ with PM_10_(lag5) model could provide a scientific basis for formulating influenza control policies and public health interventions. Timely prediction of influenza incidence is essential for effective epidemic control strategies and minimizing disease transmission risks.

## 1 Introduction

Influenza, a widely prevalent respiratory infection, exerts a substantial impact on the health of millions of people worldwide each year, leading to severe morbidity and occasional deaths (1). While, like other respiratory infections, influenza is typically most prevalent during the winter and spring seasons, recent reports have illuminated a noteworthy surge in summer influenza cases (2). This emerging trend presents fresh challenges for health authorities and influenza surveillance efforts. The onset and propagation of influenza are influenced by a multitude of factors, including environmental indicators such as air pollutants (3) and meteorological factors (4). Therefore, it is of paramount importance to attain accurate predictions of influenza incidence and develop a thorough understanding of the factors that influence it.

Timely prediction of infectious diseases is essential to maintaining and improving public health (5). It helps the government to formulate and implement effective epidemic control strategies, ensuring the availability of adequate medical resources and healthcare personnel, thereby minimizing the risk of disease transmission. Currently, various methods are employed for predicting infectious diseases, encompassing infectious disease dynamics model (6), logistic regression model (7), gray prediction theory (8, 9), ARIMA model (10–12), Prophet model (13), Holt-Winters model (14), and LSTM models (15). Each of these methods possesses its own set of advantages and drawbacks. Notably, the ARIMA model stands out in its ability to accurately identify the seasonality and trends of infectious diseases. For instance, Wu et al. utilized the ARIMA method to forecast the incidence of pulmonary tuberculosis under the regular COVID-19 epidemic prevention and control measures in China (16). Ahn et al. (17) effectively applied the ARIMA model to anticipate the incidence of rheumatic diseases during the COVID-19 pandemic in Korea. While previous studies have extensively delved into the prediction of infectious diseases, researchers often overlook the potential impacts of air pollution and meteorological factors on infectious diseases. There exists a certain degree of correlation between environmental indicators and the incidence of infectious diseases (18, 19). Thus, the inclusion of environmental indicators in the predictive model for infectious diseases is anticipated to enhance the accuracy of predictions to some extent.

In recent years, the incidence of influenza in Fuzhou has been increasing year by year, adding to the challenges of disease prevention and treatment. Notably, in 2023, during a spring peak in Fuzhou, the monthly reported cases of influenza reached 2,749, marking the highest number reported in a single month over the past decade. Therefore, the analysis of factors influencing influenza incidence and the provision of corresponding predictions and early warnings are crucial for the development of effective prevention and control strategies.

Our study initiated an analysis of the impact of environmental indicators, including air pollution and meteorological factors, on influenza incidence. It then developed an optimal ARIMA model based on influenza incidence data. Subsequently, to enhance prediction accuracy, environmental indicators were systematically introduced into the optimal ARIMA model, leading to the establishment of the ARIMAX model. Finally, we selected the optimal ARIMAX model for the prediction analysis of influenza incidence in Fuzhou.

## 2 Materials and methods

### 2.1 Study area and data sources

Fuzhou, situated in the southeast coastal area of China, serves as the capital city of Fujian Province and spans an area of 11,968.53 square kilometers. As of the end of 2022, Fuzhou had a permanent resident population of 8.448 million. The monthly data on influenza cases were sourced from the Fuzhou Center for Disease Control and Prevention. The surveillance of influenza cases followed the criteria outlined by the World Health Organization and the Chinese Center for Disease Control and Prevention for influenza-like cases. Population statistics were extracted from the Fuzhou Statistical Yearbook. We utilized monthly influenza incidence (per 100,000 populations) data spanning from January 2014 to December 2022. This dataset was split into two subsets: a training set covering the period from January 2014 to December 2022, and a test set spanning from January 2023 to September 2023.

The monthly air pollution monitoring data used in this study covers the period from January 2014 to September 2023 and was provided by the Environmental Monitoring Center under the Environmental Protection Administration of Fuzhou. The air pollutants included particulate matter 2.5 μm (PM_2.5_), particulate matter 10 μm (PM_10_), sulfur dioxide (SO_2_), carbon monoxide (CO), nitrogen dioxide (NO_2_), and ozone (O_3_). Simultaneously, the monthly meteorological data for the same period were procured from the Fuzhou Meteorological Bureau, encompassing meteorological factors such as monthly average temperature (°C), monthly maximum temperature (°C), monthly minimum temperature (°C), and monthly average wind speed (m/s). The monitoring data for the above environmental indicators was obtained with authorization from the Fuzhou Environmental Protection Bureau and the Fuzhou Meteorological Bureau.

### 2.2 Construction of the seasonal ARIMA model

Autoregressive Integrated Moving Average Model (ARIMA) is a widely-used method for the analysis and prediction of time series data (20). It finds applications in forecasting infectious diseases like varicella (21), tuberculosis (22), and COVID-19 (23). The fundamental concept underlying ARIMA model is to utilize historical data to make future predictions. ARIMA model is primarily composed of three components: Autoregressive (AR), Integration (I), and Moving Average (MA). For time series data exhibiting periodic patterns, the Seasonal Autoregressive Integrated Moving Average Model (SARIMA) combines seasonal differencing with the standard ARIMA model, making it well-suited for modeling data with recurring characteristics.

In our study, we developed a SARIMA model denoted as ARIMA(p, d, q) (P, D, Q)_s_, where p signifies the AR order, d stands for the differencing order and q represents the MA order. Meanwhile, s indicates the period of seasonal trend, while P, D and Q correspond to the seasonal terms within the SARIMA model. The determination of these parameters, (p, d, q) and (P, D, Q), is achieved through an analysis of the Partial Autocorrelation Function (PACF) and the Autocorrelation Function (ACF). The choice of the parameter s depends on the length of the seasonal cycle. The seasonal model can be mathematically represented as follows:


(1) $$\phi_{p}\left(B\right){\tilde{\phi }}_{p}\left(B^{s}\right)y_{t}^{*}=\theta_{q}\left(B\right){\tilde{\theta }}_{Q}\left(B^{s}\right)\varepsilon_{t}$$

In Equation 1, $\phi_{p}\left(B\right)$ represents a non-seasonal autoregressive lag polynomial, ${\tilde{\phi }}_{p}\left(B^{s}\right)$ represents seasonal moving average lag polynomial, $\theta_{q}\left(B\right)$ represents seasonal moving average lag polynomial. To ensure the stability of our time series, we initially applied differencing, a crucial step in the analysis. We then conducted an augmented Dickey–Fuller (ADF) test to verify the temporal stability of the series. Subsequently, we employed the corrected Akaike’s information criterion (AICc) to assess the goodness of fit of the SARIMA model, with the model associated with the lowest AICc value considered the optimal choice. Finally, we conducted the Ljung–Box test to ascertain whether the residual sequence of the model exhibited characteristics of white noise. If the *p*-value is greater than 0.05, the model satisfies the test’s criteria and can be employed for predictive analysis.

### 2.3 Construction of the ARIMAX model

ARIMAX model, which incorporates exogenous variables related to the target time series as input variables, builds upon the foundation of the ARIMA model to enhance prediction accuracy (24). The primary objective of the ARIMAX model is to capture trends and seasonal fluctuations within time series data by amalgamating autoregressive, differencing, moving average components, and exogenous variables, thereby offering precise predictions and robust analytical capabilities. In contrast to the ARIMA model, the ARIMAX model takes into account exogenous variables that are associated with the time series data. These exogenous variables can encompass other time series data or non-time series data, such as environmental indicators (25, 26) and government policies (27). The role of exogenous variables is to furnish additional information that aids in refining model fitting and prediction accuracy.

In this study, we developed an ARIMAX model for each exogenous environmental variable using data from six air pollutants and four meteorological factors. Our approach consisted of three main steps: Initially, we conducted the cross-correlation function (CCF) to assess the time-delay correlation between different variables and influenza incidence. Subsequently, we integrated significant environmental indicators as exogenous variables into the optimal ARIMA model, thereby creating alternative ARIMAX models. Finally, we selected the best-fitting ARIMAX model based on three criteria: (a) the Akaike Information Criterion (AIC), Corrected Akaike Information Criterion (AICc), Bayesian Information Criterion (BIC), Root mean squared error (RMSE) values are smaller than the optimal ARIMA model; (b) the degree that the residual sequence of the model is white noise by Ljung-Box test; (c) the model’s performance in predicting influenza incidence in 2023.

The primary innovation of our study lies in the integration of environmental indicators into the ARIMAX framework. By incorporating exogenous variables related to influenza incidence, we can gain a more comprehensive understanding of the multifaceted factors influencing disease transmission. This approach not only improves the accuracy of our predictions but also provides valuable insights for public health interventions. Furthermore, we employ advanced model selection criteria, such as the corrected AICc, to ensure optimal model fitting. Through these enhancements, our research contributes a novel perspective to the application of ARIMA models in the field of epidemiology, demonstrating their adaptability and relevance in addressing contemporary public health challenges.

### 2.4 Statistical methods

Descriptive statistics were employed to illustrate changes in influenza incidence, air pollutants and meteorological factors. Time series plots (line plots) were utilized to visualize their temporal distribution. The cross-correlation function (CCF) was used to evaluate the lag effect of environmental influencing factors. For the development of ARIMA and ARIMAX models, as well as data visualization, we utilized the R packages “forecast,” “stats,” and “ggplot2” in R (version 4.2.1, The R Foundation). The significance level was set at 0.05.

### 2.5 Ethical approval and consent to participate

We obtained ethical approval from the Ethical Review Committee of the Fuzhou Center for Disease Control and Prevention (Approval No. IRB2020008) to conduct a secondary analysis of aggregated data collected by the Fuzhou CDC, China. The informed consent requirement was waived by the Ethical Review Committee of the Fuzhou Center for Disease Control and Prevention for this study. This study was carried out following the Helsinki Declaration contents.

## 3 Results

**Figure 1:**
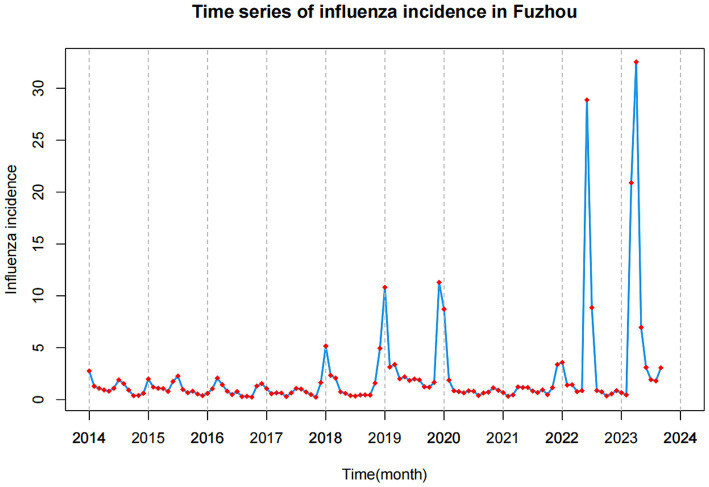
Time series of influenza incidence in Fuzhou from January 2014 to September 2023.

From January 2014 to September 2023, a total of 21,573 cases of influenza were reported in Fuzhou, with an incidence rate of 2.228 ± 4.593 (as shown in Table 1). The highest number of cases was recorded in June 2023, with 2,749 reported cases. Analysis of the time series chart of influenza incidence reveals that the peak of influenza cases typically occurs around January each year. Overall, there is a noticeable year-by-year increase in influenza incidence (as depicted in Figure 1).

**Table 1:** The descriptive statistics of the monthly influenza incidence and environmental indicators in Fuzhou, 2014–2023.

Variable	Range	Mean ± S.D.	P25	P50	P75	IQR
Incidence (/100, 000)	0.236–32.540	2.228 ± 4.593	0.645	0.974	1.845	1.200
Average temperature (°C)	9.528–30.000	19.893 ± 6.114	13.926	20.000	25.831	11.905
Maximum temperature (°C)	12.050–35.000	23.600 ± 6.346	17.540	23.140	29.230	11.690
Minimum temperature (°C)	6.291–25.000	16.182 ± 5.923	10.059	16.390	21.960	11.901
Average wind speed (m/s)	4.300–9.681	6.762 ± 0.985	6.112	6.700	7.336	1.224
PM_2.5_ (μg/m^3^)	12.000–56.000	24.160 ± 7.912	18.000	23.000	29.000	11.000
PM_10_ (μg/m^3^)	23.000–89.000	45.620 ± 12.901	36.000	44.000	53.000	17.000
SO_2_ (μg/m^3^)	3.000–16.000	5.479 ± 1.827	4.000	5.000	6.000	2.000
CO (mg/m^3^)	0.326–1.165	0.660 ± 0.142	0.577	0.668	0.735	0.158
NO_2_ (μg/m^3^)	8.000–52.000	24.060 ± 8.915	17.000	23.000	30.000	13.000
O_3_ (μg/m^3^)	45.000–130.000	88.260 ± 18.542	75.000	87.000	102.000	27.000

**Figure 2:**
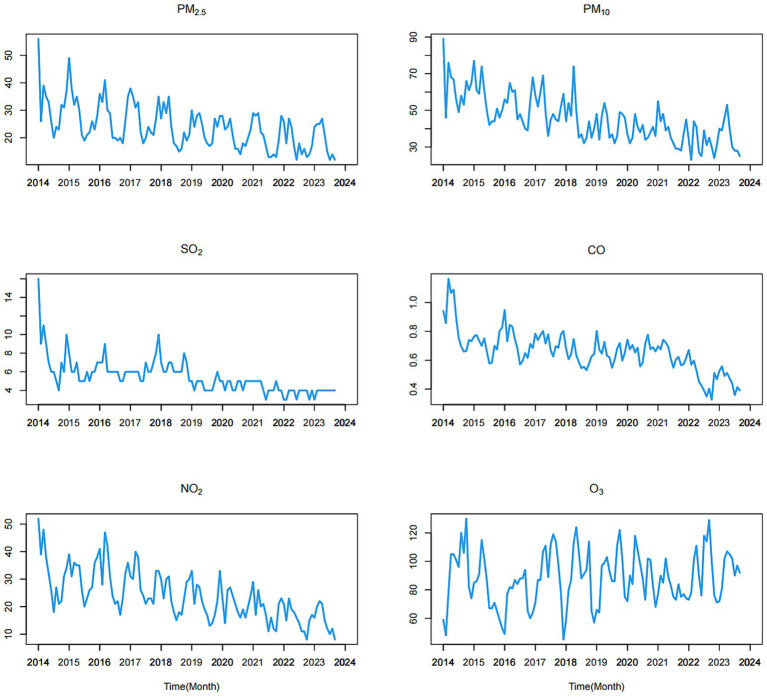
Time series of the six air pollution variables from January 2014 to September 2023.

Upon reviewing the data from the past few years, it becomes evident that nearly every winter is marked by severe air pollution in Fuzhou. Simultaneously, there is a notable increase in the incidence of influenza. Overall, the concentrations of all other five air pollutants, with the exception of O_3_, exhibit a consistent downward trend, as illustrated in Figure 2. The mean concentrations of PM_2.5_, PM_10_, SO_2_, CO, NO_2_, and O_3_ were 24.160, 45.620, 5.479, 0.660, 24.060, and 88.260 μg/m^3^, respectively.

**Figure 3:**
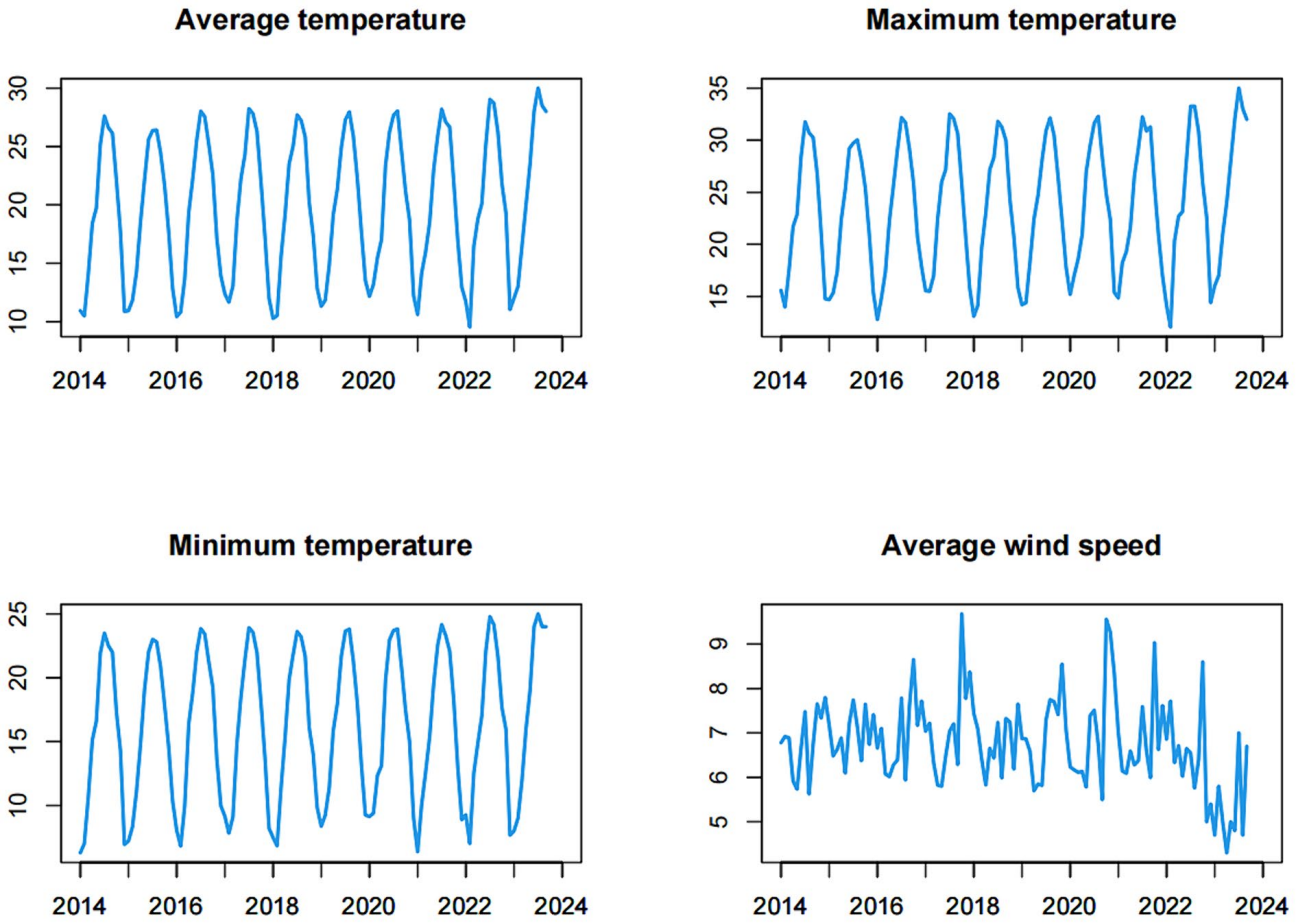
Time series of the meteorological factors (monthly average temperature, monthly maximum temperature, monthly minimum temperature, monthly average wind speed) from January 2014 to September 2023.

During the study period, the time series of meteorological factors exhibited a strong cyclical and seasonal pattern overall, with peak values occurring during the summer and troughs observed in the winter (as depicted in Figure 3). The mean values of the monthly average temperature, maximum temperature, minimum temperature and average wind speed were 19.893, 23.600, 16.182, and 6.762 m/s, respectively.

We investigated the lagged relationship between 10 environmental indicators and influenza incidence using cross-correlation analysis. As illustrated in Table 2, SO_2_, CO, NO_2_, average temperature, maximum temperature, and minimum temperature exhibited direct and statistically significant associations with influenza incidence, while the lag variables for the other three environmental indicators also displayed significant associations with influenza incidence.

**Table 2:** The correlation coefficients and maximum lag correlation coefficients between influenza incidence and environmental indicators.

	PM_2.5_	PM_10_	SO_2_	CO	NO_2_	O_3_
Corr-Coef	−0.018	−0.070	−0.183*	−0.220*	−0.183*	−0.006
Max lag Corr-Coef	−0.266*	−0.291*	−0.184*	−0.326*	−0.259*	0.238*
Its lag order (Max)	5	5	1	4	4	3

	Ave.temp	Max.temp	Min.temp	Ave.ws
Corr-Coef	−0.053*	−0.051*	−0.054*	−0.240
Max lag Corr-Coef	0.211*	0.225*	0.195*	−0.290*
Its lag order (Max)	3	3	3	2

*p < 0.05; Ave.temp, average temperature; Max.temp, maximum temperature; Min.temp, minimum temperature; Ave.ws, average wind speed.

**Figure 4:**
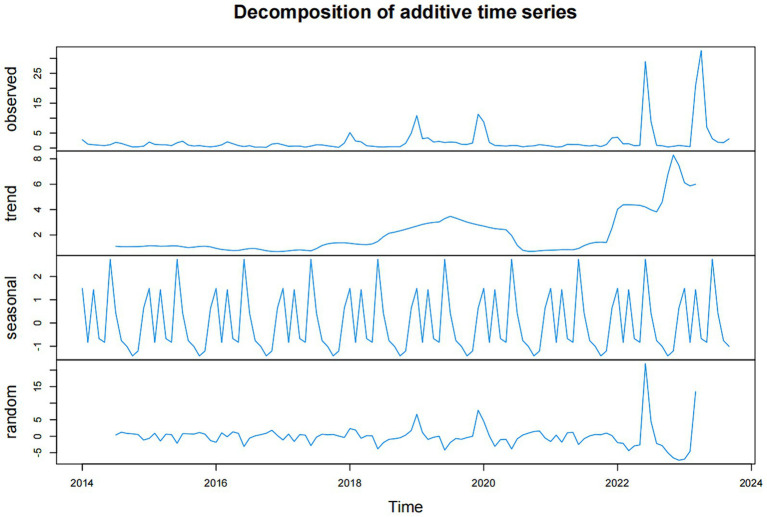
The data of influenza incidence in Fuzhou were decomposed into trend part, seasonal part and random part.

To begin with, it is imperative to establish an optimal ARIMA model for predicting influenza incidence in Fuzhou. Prior to modeling, we conducted an ADF test to assess the stability of both influenza incidence and 10 environmental indicators, aiming to ascertain if differential processing was necessary. All *p*-values from the tests were found to be less than 0.05, signifying the data were stationary and did not need to be differential processed. Consequently, we conclude that the parameters d and D in the ARIMA(p, d, q) (P, D, Q)_s_ model were both 0. Given that our predictive models were constructed using influenza incidence data spanning January 2014 to December 2022, we decomposed the data into trend, season, and random items. The influenza time series showed an upward trend. Meanwhile, this analysis also revealed a pronounced seasonality in influenza incidence data, characterized by a seasonal period of 12 (refer to Figure 4). Consequently, the parameter s of the ARIMA model was set at 12, and the model can be expressed as ARIMA(p, 0, q) (P, 0, Q)_12_.

**Figure 5:**
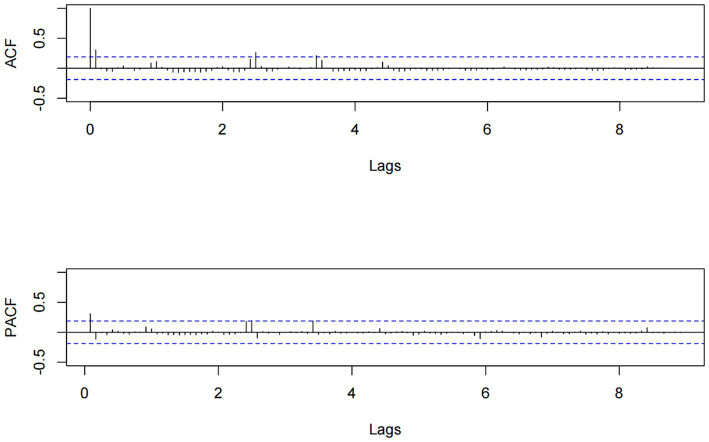
The ACF and PACF charts for influenza incidence data from January 2014 to December 2022.

We developed the model using data from the training set (January 2014 to December 2022) and assessed the prediction performance of the model using the test set data (January 2023 to September 2023). To determine the values of the remaining ARIMA model parameters p, q, P, and Q, we generated ACF and PACF plots based on the training set data. The plots for ACF and PACF reveal the temporal dependence of influenza incidence, with maximum autocorrelation and partial correlation coefficients observed at lags 0 (refer to Figure 5).

Through the analysis of the ACF and PACF plots of the original time series, it can be determined that the remaining parameters p, q, P, and Q of the ARIMA model should be 0, or 1. To automatically identify the model order of the ARIMA model, we used the auto.arima function from the “forecast” package to select a total of 13 alternative models (Table 3). Finally, the optimal model was identified as ARIMA(0, 0, 1) (1, 0, 0)_12_, boasting the lowest AIC, AICc, and BIC values, which stood at 552.910, 553.303, and 563.640, respectively. Additionally, the Ljung–Box test confirmed that the residual sequence resembles white noise (*p* > 0.05). The ARIMA(0, 0, 1) (1, 0, 0)_12_ model excelled in both fitting and predicting influenza incidence. When applied to the training set, the model yielded the fitting RMSE of 3.002; the model was employed to predict influenza incidence in the test set, achieving the predicting RMSE of 12.475.

**Table 3:** Parameters and AICc of the alternative ARIMA models.

Alternative ARIMA model	AICc
ARIMA(2,0,2) (1,0,1)_12_	Inf
ARIMA(1,0,0) (1,0,0)_12_	554.387
ARIMA(0,0,1) (0,0,1)_12_	553.402
ARIMA(0,0,1) (1,0,1)_12_	555.492
ARIMA(0,0,1) (0,0,2)_12_	555.519
**ARIMA(0,0,1) (1,0,0)12**	**553.303**
ARIMA(0,0,1) (2,0,0)_12_	555.493
ARIMA(0,0,1) (2,0,1)_12_	Inf
ARIMA(0,0,0) (1,0,0)_12_	562.445
ARIMA(1,0,1) (1,0,0)_12_	555.499
ARIMA(0,0,2) (1,0,0)_12_	555.499
ARIMA(1,0,2) (1,0,0)_12_	Inf
ARIMA(0,0,1) (1,0,0)_12_	560.726

The bold values represent the best performing models and parameters. Inf, Infinity.

To investigate the potential influence of environmental indicators, such as air pollutants and meteorological factors, on influenza incidence, we systematically integrated these environmental indicators one by one into the ARIMA(0, 0, 1) (1, 0, 0)_12_ model to formulate an optimal ARIMAX model. We integrated the maximum lag correlation variables for each environmental indicator into the ARIMA(0, 0, 1) (1, 0, 0)_12_ model, thus creating 10 distinct ARIMAX models. The Ljung–Box test was employed to assess these 10 models, and results indicated that the residual sequences of the models exhibited white noise characteristics (All *p* > 0.05).

Based on the outcomes summarized in Table 4, it was determined that the ARIMAX(0, 0, 1) (1, 0, 0)_12_ with PM_10_(lag5) model had the lowest AIC, AICc, and BIC values, signifying superior fitting accuracy and suitability for predicting influenza incidence in Fuzhou. During the model-fitting phase using the training aset, this ARIMAX model achieved a RMSE of 2.999. When applied to forecast influenza incidence in the test set, the model had an RMSE of 12.033.

**Table 4:** The performance of the ARIMA(0, 0, 1) (1, 0, 0)_12_ and 10 ARIMAX models.

Model	Variable	MA(1)	SAR(1)	AIC	AICc	BIC
ARIMA(0,0,1) (1,0,0)_12_		0.503*	0.387*	552.910	553.300	563.640
ARIMA(0,0,1) (1,0,0)_12_ with PM_2.5_(lag5)	−0.048*	0.516*	0.401*	533.560	534.170	546.730
ARIMA(0,0,1) (1,0,0)_12_ with PM_10_(lag5)	−0.068*	0.520*	0.426*	**529.740**	**530.360**	**542.910**
ARIMA(0,0,1) (1,0,0)_12_ with SO_2_(lag4)	−0.084*	0.500*	0.387*	550.580	551.180	563.950
ARIMA(0,0,1) (1,0,0)_12_ with NO_2_(lag4)	−0.083	0.499*	0.388*	535.580	536.190	548.800
ARIMA(0,0,1) (1,0,0)_12_ with CO(lag4)	−4.254	0.503*	0.399*	536.560	537.170	549.780
ARIMA(0,0,1) (1,0,0)_12_ with O_3_(lag3)	0.031	0.515*	0.396*	539.990	540.600	553.260
ARIMA(0,0,1) (1,0,0)_12_ with Ave.temp(lag3)	0.007*	0.519*	0.400*	542.630	543.240	555.900
ARIMA(0,0,1) (1,0,0)_12_ with Max.temp(lag3)	0.023*	0.516*	0.397*	542.550	543.160	555.820
ARIMA(0,0,1) (1,0,0)_12_ with Min.temp(lag3)	−0.012*	0.524*	0.405*	542.620	543.220	555.890
ARIMA(0,0,1) (1,0,0)_12_ with Ave.ws(lag2)	−0.067	0.521*	0.387*	546.680	547.280	560.000

*P < 0.05; Ave.temp, average temperature; Max.temp, maximum temperature; Min.temp, minimum temperature; Ave.ws, average wind speed. The bold values represent the best performing models and parameters.

**Figure 6:**
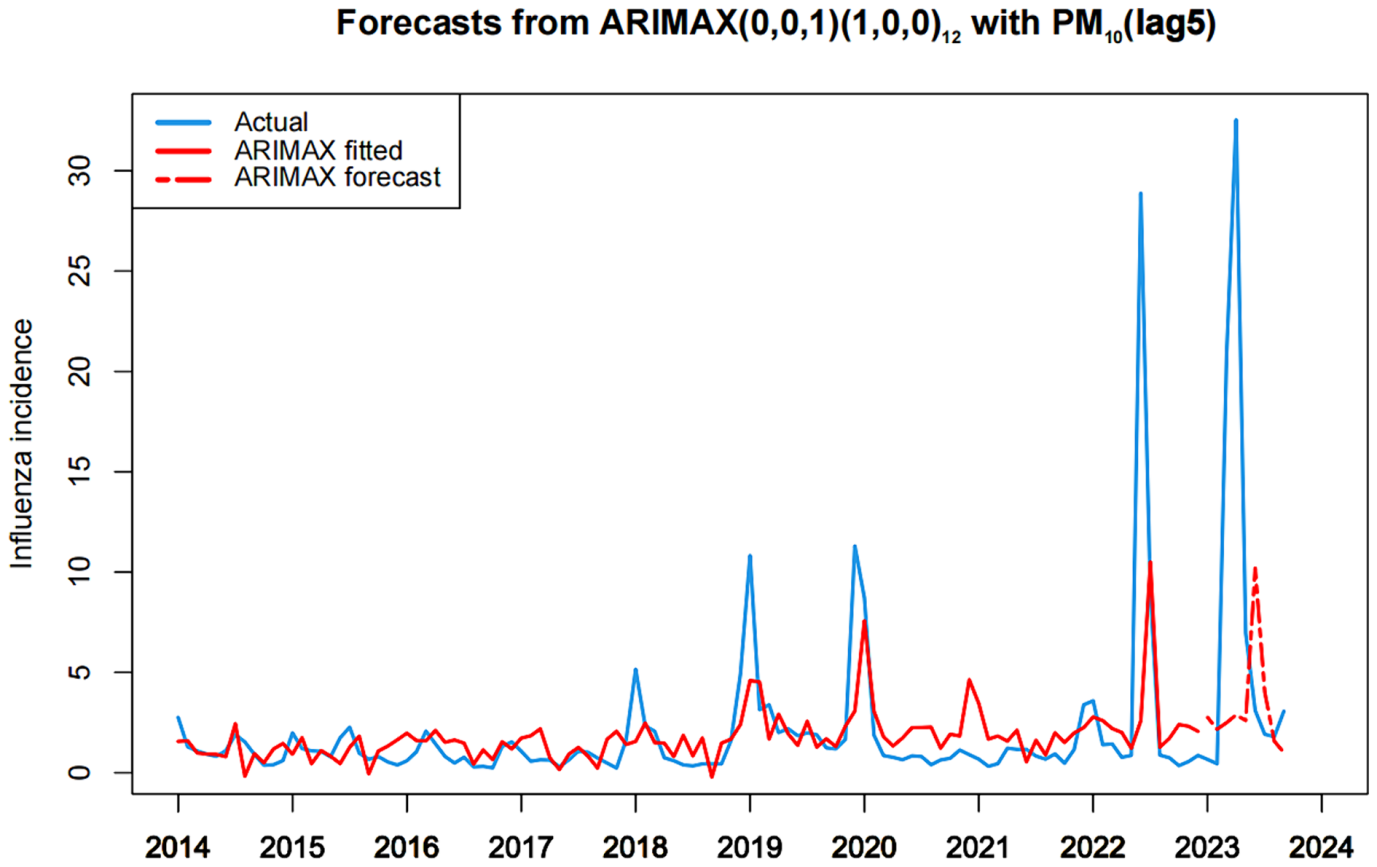
Chart of fitting and predicting influenza incidence based on ARIMAX (0,0,1)(1,0,0)_12_ with PM_10_.

Figure 6 graphically presents the fitting and predictive results of influenza incidence rates based on the ARIMAX(0, 0, 1) (1, 0, 0)_12_ with PM_10_(lag5) model. These results demonstrate the efficacy of the ARIMAX(0, 0, 1) (1, 0, 0)_12_ with PM_10_(lag5) model in accurately forecasting influenza incidence in Fuzhou. Notably, the model displayed commendable fitting accuracy in both the training and test sets.

## 4 Discussion

Influenza is a respiratory viral disease caused by the influenza virus (28). It typically manifests with acute respiratory symptoms, but for individuals with weaker immune systems, such as the young, the older adults, or those with compromised immunity, influenza can lead to more severe complications even life-threatening outcomes (2). Over the past decade, Fuzhou has witnessed a notable surge in the incidence of influenza, indicating a critical influenza epidemic. Hence, investigating the factors influencing influenza incidence is crucial for the evidence-based development of influenza control policies and the implementation of timely public health interventions.

In 2022, the winter flu peak did not occur in Fuzhou, primarily attributed to the outbreak of COVID-19 and the strict epidemic prevention and control measures implemented, including the complete suspension of in-room dining and the promotion of remote work. These measures effectively reduced interpersonal contact, thereby mitigating the spread of influenza. The proactive interventions in response to the COVID-19 outbreak in Fuzhou had a positive impact on curbing the high incidence of influenza. However, China removed many restrictive COVID-19 prevention and control measures after January 8, 2023. It resulted in a rapid increase in COVID-19 infections and necessitated home-based treatments for many citizens, contributing to a partial reduction in the spread of influenza. These observations underscore the need for in-depth analysis in future studies to understand the specific mechanisms and long-term trends of various public health interventions on influenza transmission.

During the period from 2022 to 2023, Fuzhou experienced consecutive summer influenza peaks, with a higher number of reported cases in both years. Apart from the conducive climate conditions of high temperature and humidity during summer, which potentially facilitate the transmission of the influenza virus, the reasons behind the summer influenza peaks in the 2 years might be different, contingent upon the contextual circumstances prevailing at the time.

In 2022, amidst a significant influenza pandemic, Fuzhou encountered no COVID-19 outbreak in June 2022, and residents reduced their mask-wearing behavior due to hot weather conditions. Concurrently, with medical resources extensively allocated for monitoring and treating respiratory diseases during the influenza pandemic, this likely resulted in intensified surveillance and reporting of influenza cases. During June to July 2023, the emergence of a summer influenza peak in Fuzhou may be associated with China’s relaxation of numerous restrictive COVID-19 control measures, such as mask-wearing and avoidance of crowded places, effective from January 8, 2023. Subsequently, residents’ immune systems may have weakened. During the COVID-19 pandemic, heightened attention to personal protection and hygiene practices might have reduced exposure of the immune system to common viruses. Following the easing of restrictions, resumption of social activities may have diminished the immune system’s resistance to the influenza virus, thereby precipitating its outbreak. Moreover, there was a notable increase in social gatherings. Post-lockdown, individuals likely resumed more social and congregational activities such as dining, gatherings, and tourism. Such congregation could have facilitated the spread of the influenza virus, contributing to the peak in influenza cases. Finally, the relaxation of healthcare resource pressures could also have played a role. During the COVID-19 pandemic, medical resources were primarily directed toward combating the outbreak, potentially leading to neglect in the prevention and control of other diseases. Post-lockdown, while healthcare resources might have eased, reduced vigilance toward COVID-19 may have led to diminished attention and control measures for influenza, thereby fostering its transmission.

There have been many previous studies have demonstrated the association between various diseases and environmental indicators, including diseases like dengue fever (29, 30), COVID-19 (31–33), and tuberculosis (34). In the case of influenza, environmental indicators can influence the occurrence of influenza epidemics through factors such as the variation and transmission of influenza virus and the immune status of the population (35). The Cross-Correlation Function (CCF) measures the correlation between two variables at different time lags, making it particularly well-suited for analyzing lagged effects and time-delayed relationships between variables. Additionally, as the impact of environmental indicators may exhibit a time lag in disease incidence (36, 37), we investigated the lagged correlation between influenza incidence and these environmental indicators.

Our analysis revealed that most of the lagged air pollution variables exhibited a negative association with influenza incidence. This implies that as air pollution levels increase, the incidence of influenza tends to decrease. This negative correlation can, in part, be attributed to the adverse impact of severe air pollution on the human immune system, thereby increasing the risk of infectious diseases (38). However, the manifestation of this weakened immune system in terms of influenza incidence may not be immediately evident and could require some time to become apparent. This phenomenon might also be linked to public awareness of declining air quality. Following the perception of deteriorating air quality, individuals may have adopted proactive protective measures, including reducing outdoor activities and wearing face masks to mitigate their exposure to air pollution (39). These self-protective behaviors could contribute to a reduction in the likelihood of influenza virus transmission, consequently lowering the incidence of influenza. Moreover, it’s essential not to overlook the impact of the COVID-19 pandemic in recent years. From 2019 to 2022, widespread mask-wearing in public to prevent COVID-19 not only effectively curtailed the spread of the novel coronavirus but also had the side effect of reducing the transmission of influenza (40). Interestingly, our analysis showed a positive association between the third-order lagged variable of O_3_ and influenza incidence. This positive correlation may be attributed to high concentrations of O_3_ inducing lung inflammation (41), which weakens the immune system and heightens susceptibility to infections. Furthermore, O_3_ might also influence the pathogen’s transmission mode, potentially rendering it more prone to airborne transmission.

The analysis revealed that influenza incidence demonstrated a negative association with three distinct temperature variables, indicating that the higher the temperature, the lower the influenza incidence. The intricacies of this relationship become more pronounced when accounting for the temperature’s delayed effects. The third-order lagged temperature variable demonstrated a significant positive correlation with influenza incidence. This observed pattern could be indicative of the seasonal pattern of influenza virus transmission, further complicated by temperature’s influence on human behavior and immune responses. The transmission of the influenza virus may exhibit nuanced seasonal variations, influenced by changing atmospheric temperatures (42). While increasing temperatures generally correlate with reduced influenza incidence, the full manifestation of this trend may experience delays due to the time-sensitive nature of human immune and behavioral adjustments. This suggests that people may still be at risk of spreading the flu virus for some time after the temperatures rise. Notably, behavioral patterns also shift in response to seasonal temperature changes. During warmer periods, increased outdoor activities and social interactions could inadvertently amplify influenza transmission risks, potentially leading to a spike in cases as temperatures rise. In relation to average wind speed, while the mean value demonstrated no significant correlation with influenza, the second-order lagged wind speed showed a significant negative correlation with influenza incidence, indicating that wind speed also has a long-term lag negative correlation effect on influenza incidence.

We utilized time series analysis to examine the correlation between influenza incidence and environmental indicators in Fuzhou from January 2014 to September 2023. The environmental indicators encompassed air pollution variables (PM_2.5_, PM_10_, SO_2_, CO, NO_2_, and O_3_) and meteorological factors (mean temperature, minimum temperature, maximum temperature, and wind speed). In our study, the time series data of influenza incidence in Fuzhou from January 2014 to September 2023 were found to be stationary and exhibited seasonal distribution. However, since the model used in the study was able to effectively capture the seasonal effects, there was no need to difference the time series data of influenza incidence. We also experimented with introducing seasonal differences in the time series data of influenza incidence; however, we observed that this adjustment did not lead to an improvement in the model’s performance. Therefore, the data of influenza incidence were not processed by differencing in this study. First, the ARIMA(0, 0, 1) (1, 0, 0)_12_ model was identified as the most optimal ARIMA model for forecasting influenza incidence in Fuzhou, with AIC, AICc, and BIC values of 552.910, 553.300, and 563.640, respectively. This model was employed to fit the training set, yielding a fitting RMSE of 3.002. Subsequently, the model was utilized for prediction analysis on the test set, yielding a predicting RMSE of 12.475. To enhance prediction accuracy, the maximum lag correlation variables of environmental indicators during the study period were incorporated into the optimal ARIMA model. The results demonstrated that the AIC, AICc, and BIC values of the 10 ARIMAX models, each including a single environmental index, were lower than those of the ARIMA(0, 0, 1) (1, 0, 0)_12_ model. This suggested that considering environmental indicators could enhance the predictive performance of the model. Comparing the AIC, AICc, and BIC values of all ARIMAX models, the ARIMAX(0, 0, 1) (1, 0, 0)_12_ with PM_10_(lag5) model had the lowest AIC, AICc, and BIC values, specifically 529.740, 530.360, and 542.910, respectively. Moreover, this model exhibited a fitting RMSE of 2.999 and a predicting RMSE of 12.033, both of which were superior to the optimal ARIMA model. The ARIMAX(0, 0, 1) (1, 0, 0)_12_ with PM_10_(lag5) model can be effectively employed for short-term prediction of influenza incidence in Fuzhou. This approach provides a scientifically grounded basis for formulating influenza control policies and public health interventions in Fuzhou.

The findings from our study suggest several implications for further research. Firstly, there is a need to explore the specific mechanisms through which environmental factors, such as air pollution and meteorological conditions, influence influenza transmission dynamics. Additionally, future studies could investigate the applicability of the ARIMAX model in different geographical contexts and for other infectious diseases. Expanding the dataset to include more diverse populations and environmental conditions could enhance the robustness of predictive models. Lastly, interdisciplinary research integrating public health, environmental science, and epidemiology will be essential for developing comprehensive strategies to mitigate the impact of influenza and improve public health preparedness.

In our study, we examined both ARIMA and ARIMAX modeling approaches to analyze influenza incidence in Fuzhou. The strengths of the ARIMA model include its simplicity and strong theoretical foundation, making it effective for stationary time series data. However, it does not account for external factors, which can limit its explanatory power. On the other hand, the ARIMAX model allows for the incorporation of exogenous variables, enhancing predictive accuracy and capturing lagged effects, which is crucial for understanding the impact of environmental indicators. Nevertheless, the ARIMAX model introduces complexity and relies heavily on the quality of data for the exogenous variables, which can pose challenges in interpretation and model validation. Ultimately, the ARIMAX model provided a more comprehensive analysis for our research questions. While the ARIMAX(0, 0, 1) (1, 0, 0)_12_ with PM_10_(lag5) model incorporating environmental indicators provides valuable insights into the relationship between these factors and influenza incidence, it is essential to acknowledge its limitations. Firstly, the model relies heavily on historical data, which may not capture sudden changes in environmental conditions or emerging infectious disease patterns. Additionally, while environmental indicators such as air pollution and meteorological factors are significant, they are not the sole determinants of influenza occurrence. Biological factors, human behavior, and public health interventions also play crucial roles. Thus, while our statistical analysis demonstrates a correlation, it does not imply causation, and the model’s predictions should be interpreted with caution. Therefore, while our findings suggest a potential relationship, further research, including controlled studies and experimental designs, is necessary to establish definitive causal links between environmental pollution factors and influenza incidence. Additional, future research should consider integrating biological and socio-economic factors to enhance the comprehensiveness of predictive models.

## 5 Conclusion

The incidence of influenza in Fuzhou has shown a significant increase in the past decade. Our study indicates that air pollution and meteorological factors exert an impact on influenza incidence, often exhibiting a lag effect. The ARIMAX(0, 0, 1) (1, 0, 0)_12_ with PM_10_(lag5) model was developed using historical data on influenza incidence and air pollutant levels in Fuzhou, demonstrated excellent predictive performance for forecasting influenza incidence. Therefore, the ARIMAX(0, 0, 1) (1, 0, 0)_12_ with PM_10_(lag5) model could provide a scientific basis for the formulation of influenza control policies and public health interventions in Fuzhou.

## Data Availability

The raw data supporting the conclusions of this article will be made available by the authors, without undue reservation.
